# Serial Position Markers in Space: Visuospatial Priming of Serial Order Working Memory Retrieval

**DOI:** 10.1371/journal.pone.0116469

**Published:** 2015-01-22

**Authors:** Maya De Belder, Elger Abrahamse, Mauro Kerckhof, Wim Fias, Jean-Philippe van Dijck

**Affiliations:** Department of Experimental Psychology, University of Ghent, Ghent, Belgium; Centre de Neuroscience Cognitive, FRANCE

## Abstract

Most general theories on serial order working memory (WM) assume the existence of position markers that are bound to the to-be-remembered items to keep track of the serial order. So far, the exact cognitive/neural characteristics of these markers have remained largely underspecified, while direct empirical evidence for their existence is mostly lacking. In the current study we demonstrate that retrieval from verbal serial order WM can be facilitated or hindered by spatial cuing: begin elements of a verbal WM sequence are retrieved faster after cuing the left side of space, while end elements are retrieved faster after cuing the right side of space. In direct complement to our previous work—where we showed the reversed impact of WM retrieval on spatial processing—we argue that the current findings provide us with a crucial piece of evidence suggesting a direct and functional involvement of space in verbal serial order WM. We outline the idea that serial order in verbal WM is coded within a spatial coordinate system with spatial attention being involved when searching through WM, and we discuss how this account can explain several hallmark observations related to serial order WM.

## INTRODUCTION

Working memory (WM) is a fundamental cognitive function and refers to the brief maintenance of information in an active and accessible state such that operations can be performed on it. It is considered to be crucial for major cognitive skills like language, reasoning and learning, not in the least for its major feature of maintaining serial order across multiple items (e.g., [1]). In this study, we address the particular nature of *verbal serial order WM*.

The question how the brain deals with serial order processing in WM has a long research tradition (for a review see [2]). These research efforts have resulted in several sophisticated (computational) models and theories. In general, it can be stated that the most prominent models in this domain are built on the idea that serial order coding in WM is achieved by binding the various items to-be-maintained to specific *position markers* (e.g., begin vs. end items [3]; encoding strength [4]; oscillatory response [5]; magnitude codes [6]). Despite their (relative) success in accounting for several empirical observations, these models are largely formulated on theoretical grounds and few specifications have been provided with respect to the cognitive and/or neural nature of these position markers (but see [6]). Importantly, direct empirical evidence for the existence of (any of the proposed) position markers is sparse [7].

Recently a new idea to account for serial order coding in WM was proposed—but not further developed—by Oberauer ([8]; p. 53) who suggested that a “spatial medium of representation [is used] as a projection screen for relations on nonspatial dimensions”—such as serial order. In a recent paper, we further developed this into what we refer to as the *mental whiteboard hypothesis*: (I) The position markers that provide multi-item WM with a serial context should be understood as coordinates within an internal, spatially defined system; (II) internal spatial attention is involved in searching through the resulting serial order representation; and (III) retrieval corresponds to selection by spatial attention [9]. We hereby assume that the spatial coding of serial order spontaneously occurs from left to right on the basis of the typically observed leftward bias in spatial processing [10, 11] and/or a shaping by reading direction (cf. [12, 13, 14]). Here we zoom in on the empirical foundation of this account, specifically for the verbal domain. To demonstrate a functional involvement of spatial processing in verbal serial order, the empirical foundation should be at least twofold:

*First*, it needs to be shown that retrieval from serial order WM can modulate spatial processing. Indeed, this has been confirmed across a number of recent studies [15, 16, 17]. For example, a systematic association between the ordinal position of an item in verbal WM and the response side was observed when retrieving information from serial order WM: begin elements of a WM sequence were responded to faster with left hand responses, and end elements with right hand responses (e.g., [15, 18]). This suggests that verbal WM, in its serial aspects, is more strongly associated to space than one would anticipate. Moreover, building further on the notion that internal and external spatial attention strongly interface [19, 20], it was found that the association between serial order in WM and space can be observed already directly at the level of spatial attention: using items from serial order WM as a cue in a Posner-like cueing paradigm van Dijck et al. [16, 17] showed that retrieval of an item from WM increasingly facilitated detection of right dots as the item was positioned further towards the end of the WM sequence.

Importantly, the observation that serial order WM retrieval modulates spatial processing constitutes only part of the required support. Whereas it indicates that processes in serial order WM can elicit spatial processing, they do not necessarily evidence the functional involvement of spatial processing in serial order. For example, spatial processing might have been triggered by peripheral processes unrelated to serial order per se. To this purpose, *secondly*, it is important to consider the intriguing and complementary prediction of our mental whiteboard hypothesis that verbal serial order WM retrieval can be facilitated/hindered by the processing of external *spatial* cues. That is, left-sided spatial cues should facilitate retrieval of begin elements of the WM sequence while right-sided spatial cues should facilitate retrieval of end elements. Such observations would provide a next piece of the puzzle and bring us one step closer to confirming the intrinsic role of spatial processing in serial order WM. In the current study, we tested this prediction across two experiments.

## EXPERIMENT 1

### Procedure

The study was approved by the ethical committee of the Faculty of Psychology and Educational Sciences of Ghent University and in agreement with the Declaration of Helsinki.

Nineteen participants (all participants reached the legal age of adulthood, i.e., 18 years; age range: 20–36 years; average age: 24.26 years, SD = 3.87; 13 females; 4 left-handed) completed the study after signing an informed consent in exchange for 10 euro. Participants were individually tested in a quiet room and seated behind a 17-inch monitor at a viewing distance of approximately 50 cm. Instructions and stimuli were presented in white on a black background. A QWERTY keyboard was used to register the responses.

Participants cycled repeatedly through the same three phases: I) WM sequence presentation, II) probe detection task, and III) WM sequence verification. Phase 1 started with the self-paced central presentation of 4 successive consonants, randomly selected from the list: c, f, h, m, p, s, t and v (each 0.72° × 0.84°). The instruction was to memorize the elements of the sequence in the order of presentation. After the fourth letter, a 2500ms rehearsal interval elapsed. Subsequently, a go/no-go probe detection task was initiated (phase II). Every trial of this task started with a central fixation cross (1000ms), followed by a dot (2.9°; 150ms) appearing randomly either on the left or right side of the fixation cross, and at either a more centralized (4.6° from center) or more distant location (16° from center) on the screen. Dots appeared on distant locations in 75% of the trials, and the instructions were to *only* execute the probe detection task when the dot appeared in one of the two distant locations on the screen to induce explicit processing of spatial information.

After dot presentation, the screen remained black (50ms) after which the probe letter appeared (1000ms). The task was to press the letter “b” as fast and accurate as possible with the dominant hand when the letter belonged to the WM sequence (and the dot was previously presented at a distant location on the screen), and to refrain from responding otherwise. In the probe detection task, all letters were presented twice in random order for each cycle, resulting in 16 trials per WM sequence. After a response or the response deadline (1500ms), the screen went black and following a 1000ms inter-trial-interval (ITI) the next trial was initiated.

Finally, after sixteen trials of the go/no-go probe detection task, sequence maintenance was verified (phase III) by two subsequent statements on serial order (e.g., “Kwam C voor V?”, Dutch for “Was C preceded by V?”). These statements were composed of 2 unique pairs of consecutive WM items of which the order either corresponded or not to the WM sequence (items were vertically arranged to avoid horizontal association). Care was taken within a block that the answer to both statements was unpredictable, but that over the entire experiment, equal amounts of correct and wrong statements were presented. After responding to the two statements, participants could take a self-paced break to move on to the next to-be-remembered sequence. The complete procedure, passing through the three phases, was repeated with 32 distinct WM sequences, with each letter equally often presented across all possible serial positions of the sequence. As we were mainly interested in the effects of spatial processing per se (and not whether the effects are induced by overt of covert shifts of spatial attention), eye movements were not monitored in the current study. Moreover, it has been shown that eye movements do not typically account for dot detection performance in general [21, 22], or even for specifically the relation between serial order and space (e.g., [16]; Experiment 2), suggesting an important contribution of covert spatial attention.

### Data analysis

Mean reaction times (RTs) were computed per participant per condition for the probe detection task, and submitted to a 4 × 2 Repeated Measures ANOVA with *WM-position* (4 levels: position 1 to 4) and *Dot-location* (2 levels: left distant, right distant) as within-subject variables. An interaction between *WM-position* and *Dot-location* is predicted to indicate visuospatial priming of the verbal serial order in WM. Polynomial contrasts were calculated to investigate the presence of a linear relationship to further explore the nature of the interaction [16]. Multivariate test results for repeated measures are reported.

### Results

Trials from WM sequences which were correctly remembered (i.e., a correct serial order verification of the two statements presented during phase III of the experiment) (94% correct (SD = .09)) and correct go-trials (probe detection accuracy was 95% (SD = .04) and 98% (SD = .03) for go- and no-go trials, respectively) were considered. Mean RT was 572ms (SD = 67ms).

**Figure 1:**
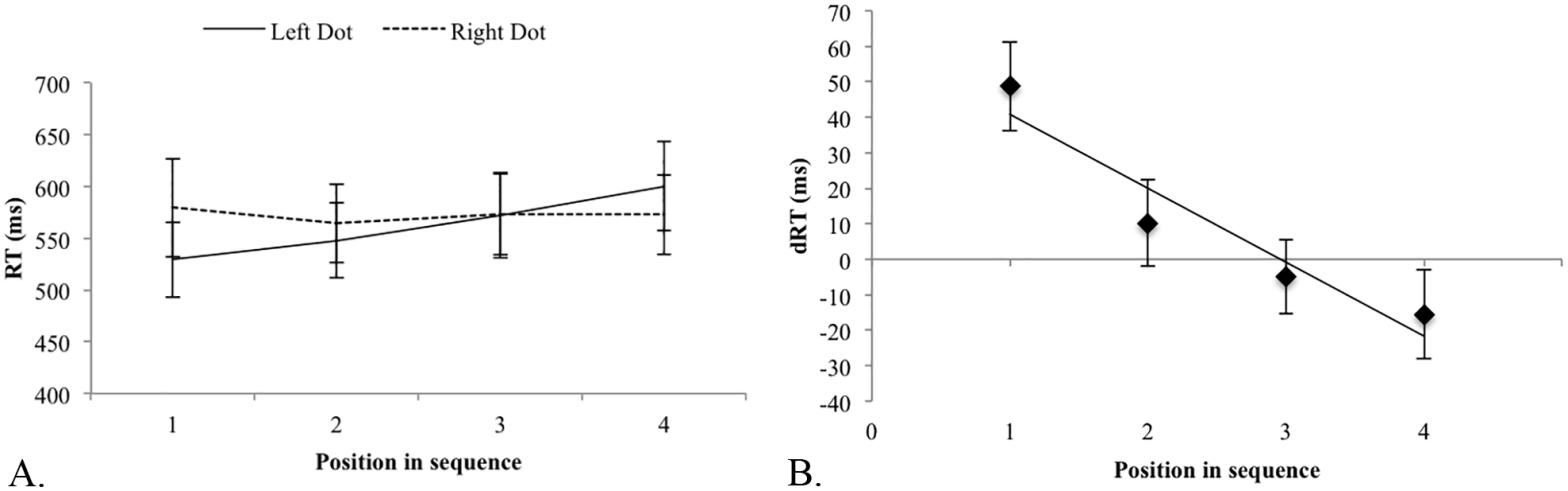
Experiment 1. A. Raw RTs for left- and right-sided dot presentations for each position in WM. The error bars indicate one standard error of the mean. B. Average RT differences between right and left-dot presentations as a function of the position in the WM sequence. Positive values indicate faster responses after dot presentation on the left side of space. The regression line reflects the linear relationship as expressed by the polynomial contrast.

The analyses revealed a main effect of *WM-position* [Wilks’ lambda = .88, F(3,16) = 5.31, *p* = .01, *η_p_* = .500]. RTs for the four different positions were 554 (SD = 66), 556 (SD = 68), 573 (SD = 77) and 587ms (SD = 78), suggesting serial scanning in WM from start to end items (cf. [16]). The *WM-position* by *Dot-location* interaction was significant [Wilks’ lambda = .91, F(3,16) = 15.09, *p* < .001, *η_p_2* = .74] (Fig. 1A), supporting the hypothesis that WM retrieval is influenced by the visuospatial primes. A polynomial contrast of *WM-position* in its interaction with *Dot-location* revealed a linear relationship [F(1,18) = 41.38, *p* < .001, *η_p_2* = .697, slope = -20.84] (Fig. 1B): the RT advantage for WM retrieval after perceiving right-sided over left-sided dots increased on average with 26ms per WM-position from start to end.

## EXPERIMENT 2

The aim of Experiment 2 was fourfold. First, we investigated whether the explicit need to process the spatial dot is a necessary condition to modulate WM retrieval or whether the mere perception of task irrelevant flashed dots is sufficient. We replicated Experiment 1, but presented task irrelevant dots unpredictably on the left or right side from the central fixation point. Second, it is known that spatial information can be encoded fast and automatically (e.g., [23]) and then quickly decays (or is actively suppressed; e.g., [24]). Therefore we manipulated the interval between dot and probe onset (100ms before, or 100 or 300ms after probe onset). Two backward prime conditions were included with the aim to maximize the chance to create overlap between serial order retrieval and spatial attention processing (cf. [25]). Third, in Experiment 1 participants could memorize the WM sequences in a self-paced fashion. However, it has been shown that encoding times can potentially impact WM (e.g., [26]). To ensure that encoding strategies did not (partly) underlie observations in Experiment 1, the presentation of WM sequences in Experiment 2 was computer-paced. Fourth, to rule out any impact of the use of the dominant hand, vocal responses were employed.

### Procedure

The study was approved by the ethical committee of the Faculty of Psychology and Educational Sciences of Ghent University and in agreement with the Declaration of Helsinki.

Twenty students (all participants reached the legal age of adulthood, i.e., 18 years; age range: 18–25 years, average age: 19.60 years (SD = 2.23); 12 females; 8 left handers) provided written informed consent and participated in exchange for course credits. Participants were seated at a viewing distance of approx. 50 cm from the computer screen. A chin-rest was used to ensure a stable viewing distance. Responses were collected with a voice-key connected to a headset microphone.

The experimental setup again involved 3 phases, but contained several differences with Experiment 1. In phase I, four letters (each 0.72° × 0.84°) pseudo-randomly sampled from the list c, f, h, k, m, p, s and v (balanced across WM positions) were sequentially presented at the center of the screen (1500ms), separated by an empty screen (200ms). Participants were instructed to memorize the stimuli in the order of presentation. After a rehearsal period (2500ms), phase II started with the presentation of a fixation cross (500ms). Two types of spatial priming were used: forward and backward priming. With forward priming, a dot (.06°; 100ms) was presented 200ms *before* probe onset (i.e., with a 100ms black screen after dot offset) appearing unpredictably on the left or right side of the screen (7.4° from center). With backward priming, the dots could appear either 100 or 300ms after probe onset. Participants were explicitly told that they could and should ignore the dots, and that they gave no useful information. Subsequently the probe letter appeared. The task was to say “JA” (Dutch for “yes”) in the microphone as fast as possible when the letter belonged to the WM sequence, and to refrain from responding otherwise. During the probe detection phase, all letters were presented once in random order. After the 1200ms probe duration or 300ms after a response, the screen went black and following an ITI of 500ms the next trial was initiated. Due to technical limitations of the voice-key device, only RTs below 901ms were recorded. Finally, after the execution of eight trials of the probe detection task, sequence maintenance was verified in phase III by similar statements on serial order as in Experiment 1. The three phases were passed through 72 times, resulting in 72 distinct WM sequences, with each letter equally often presented across all possible WM sequence positions and with trials equally balanced across experimental conditions. This resulted in 12 measurements per condition, RTs collected during the probe detection task.

### Results

Data of two participants were discarded from analyses because of low overall accuracy (2 SD below average and chance level performance on the Go trials); this mainly related to technical failure of the voice-key device (analyses including these two participants demonstrated qualitatively identical results to those described below). For the remaining participants, as in Experiment 1 only trials with correct serial order verification (84%, SD = .07) and correct go-trials (probe detection accuracy was 81% (SD = .09) and 97% (SD = .02) for the go and no-go trials, respectively) were considered. Mean RT was 519ms (SD = 60ms).

The mean RT of the different conditions was submitted to a repeated measures ANOVA with *Dot-probe interval* (DPI; 3 levels: -100, 100 and 300ms), *WM-position* (4 levels: position 1 to 4) and *Dot-location* (2 levels: left, right) as within subject variables. The multivariate test results for repeated measures are reported.

**Figure 2:**
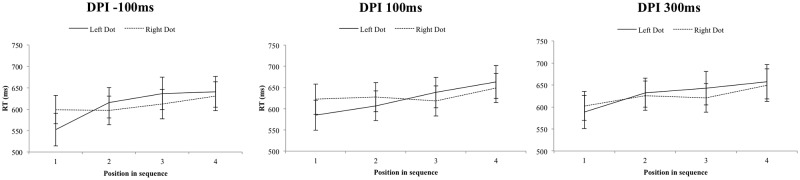
Experiment 2. The graphs display the raw RTs for left- and right-sided dot presentations for each position in WM, depending on DPI (-100ms, 100ms or 300ms). The error bars indicate one standard error of the mean, for all DPIs separately.

**Figure 3:**
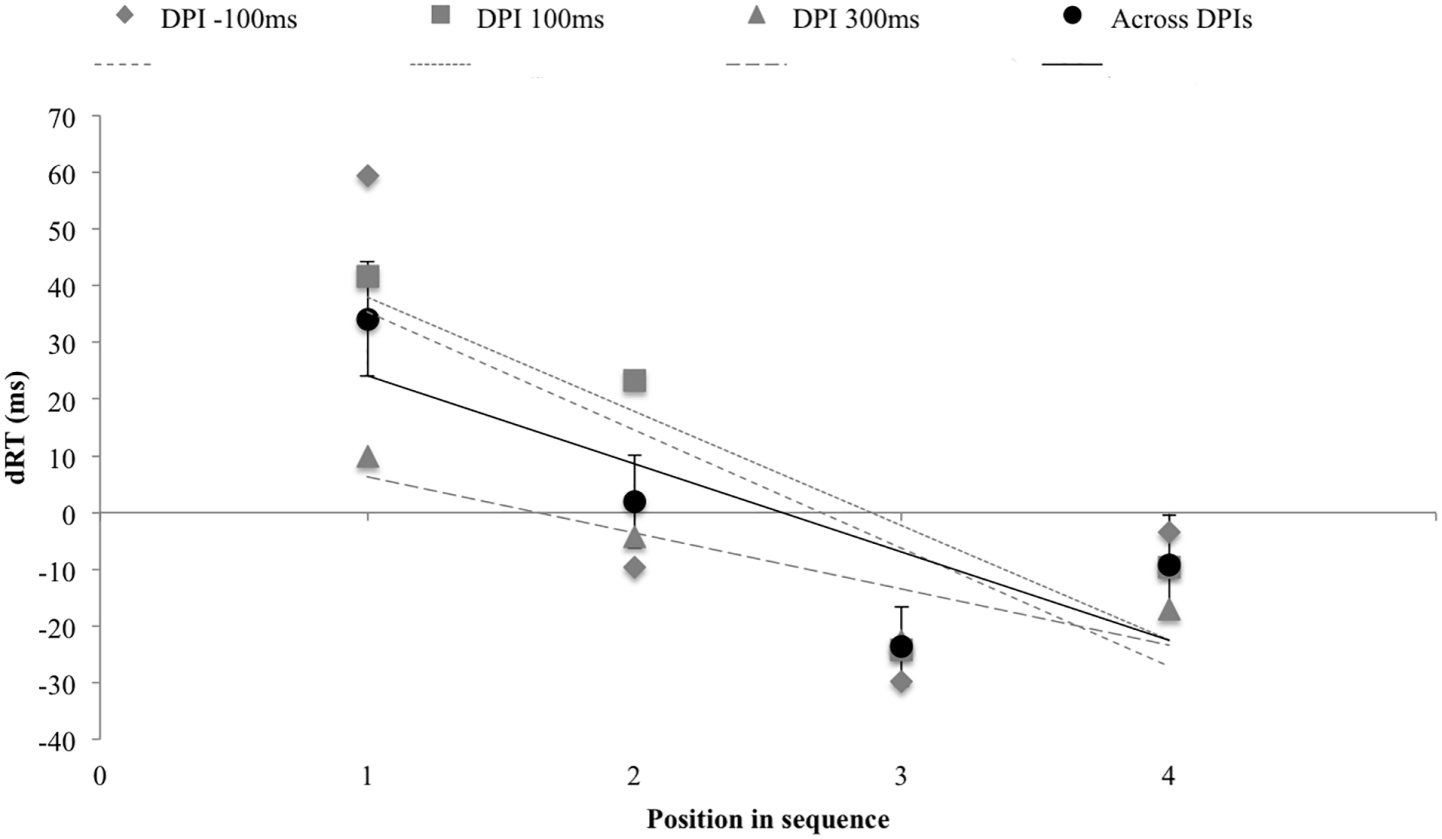
Experiment 2. The graph displays the average RT differences between right and left-dot presentations for each dot-stimulus interval as a function of the position in the WM sequence. Error bars indicate one standard error of the mean over all DPIs. Positive values correspond to faster responses after dot presentation on the left side of space. The regression line reflects the linear relationship as expressed by the polynomial contrast; where the black lines and data points display results across all DPIs and grey dashed line demonstrate this for each DPI separately.

Main effects were observed for *DPI* [Wilks’ lambda = .62, F(2,16) = 4.86, *p* = .022, *η_p_2* = .378] and *WM-position* [Wilks’ lambda = .25, F(3,15) = 14.84, *p* < .001, *η_p_2* = .748]. RTs per DPI were 514 (SD = 15.43), 529 (SD = 13.99) and 531ms (SD = 14.28); and 495 (SD = 15.13), 518 (SD = 15.46), 534 (SD = 15.22) and 551ms (SD = 13.89) per WM position. This latter main effect of WM-position suggested serial scanning of the WM sequence from start to end. The interaction between *Dot-location* and *WM-position* was significant [Wilks’ lambda = .42, F(3,15) = 6.85, p = .004, *η_p_2* = .578] (Fig. 2), and a linear polynomial contrast revealed a linear relationship [F(1,17) = 14.12, *p* = .002, *η_p_2* = .454, slope = -15.54] (Fig. 3): The advantage in RT to detect right-sided over left-sided dots increased on average with 17ms per WM position replicating the observation that a (task irrelevant) left or right-sided visuospatial prime selectively modulates the retrieval from verbal serial order WM.

All other main and interaction effects failed to reach significance, but the three-way interaction between *DPI*, *WM-position* and *Dot-location* nearly reached significance [Wilks’ lambda = .41, F(6,12) = 2.85, *p* = .058, *η_p_2* = .59], suggesting modulation in the interactions between *WM-position* and *Dot-location* based on the time of presentation of the Dot (DPI). Further two-way ANOVAs between *WM-position* and *Dot-location* for each *DPI* separately showed that a) for a dot presentation 100ms before stimulus presentation (DPI: -100) a significant interaction between *WM-position* and *Dot-location* was observed [Wilks’ lambda = .50, F(3,15) = 4.99, *p* = .01, *η_p_2* = .500], with a linear relationship [F(1,17) = 6.53, *p* = .02, *η_p_2* = .278, slope = -20.82]; b) for a dot presentation 100 after stimulus presentation (DPI: 100) a similar interaction [Wilks’ lambda = .56, F(3,15) = 3.99, *p* = .028, *η_p_2* = .444] and linear relationship were observed [F(1,17) = 10.46, *p* = .005, *η_p_2* = .381, slope = -20.12]; and c) for a DPI of 300ms no interaction was observed [Wilks’ lambda = .87, F(3,15) = .722, *p* = .554, *η_p_2* = .126]. Hence, there is weak support for the notion that irrelevant spatial information should be presented relatively close in time to the WM stimulus in order to affect WM processes.

## GENERAL DISCUSSION

The current study demonstrates that performance on serial order WM retrieval can benefit from visuospatial priming: left and right exogenous cues facilitated the retrieval of begin and end elements of a WM sequence, respectively. Whereas in Experiment 1 such an effect of the spatial cues on WM retrieval was observed when these cues were task-relevant, in Experiment 2 this facilitation also occurred spontaneously, when task-irrelevant cues were presented around the time of WM retrieval. Furthermore, the effect was observed with both manual (Experiment 1) and vocal (Experiment 2) responses, suggesting that the effect is not driven by any spatial code associated with the used effector.

In complement to previous findings of van Dijck and colleagues [15, 16, 17] the current findings affirm the *bidirectional* relation between verbal serial order WM and spatial processing as is predicted in the mental whiteboard hypothesis—which assumes intrinsic and functional involvement of spatial processing in verbal serial order WM [9]. As such, it provides a viable candidate mechanism to substantiate the more abstract notion of serial position markers in verbal serial order WM. Such markers have been a core feature of prominent serial order models (see [2] for a review), but few specifications have been proposed as their cognitive and/or neural nature (but see [6]). The current observation that external spatial cues can modulate the retrieval performance on serial order WM tasks indicates that position markers can be understood as *specific coordinates within a spatially defined system*.

Importantly, although detailed modeling is needed to understand the exact nature of cognitive dynamics, conceptually this mechanism can easily account for various hallmark observations in the domain of serial order memory [2]. First, the typical serial position effect (gradual increases in response times for items further in the sequence; cf. [27]) can be directly related to the attentional search—from left to right—through the spatially defined WM representation. Second, the observation that it is more difficult to determine the serial order for nearby compared to more distant items (i.e., distance effect [28, 29]) may be explained by the fact that in space processing, discrimination between two stimuli is more difficult when they are positioned at nearby compared to further locations (e.g., [30, 31]). Third, a similar (attentional) interference explanation may hold for the observation that errors in serial recall often involve switches between serially nearby items (i.e., transposition errors). Overall, the notion that serial order is spatially coded seems to provide a parsimonious account on the major serial order WM observations.

The perspective that verbal serial order WM is intrinsically linked to space also opens new questions to be explored. For example, it is currently unknown whether the observed involvement of space is limited to the verbal domain, or whether it reflects a property of serial order WM that is independent of the modality of the to-be-remembered items. Behaviorally, functional similarities have been observed between serial order WM for verbal and spatial items (see [32] for a review), suggesting domain-generality. This is further supported by a recent fMRI study where overlapping brain areas were recruited when processing serial order within verbal and visual WM [33]. Still, future research should directly address this question, for example by employing non-verbalizable items in paradigms such as the one used in the current study. Another outstanding question is whether the involvement of space is limited to the moment of retrieval, or whether the relation between serial order WM and space is already present during WM encoding and maintenance. Indirect evidence for the latter has recently been provided by Fischer-Baum and Benjamins [34]. They showed that the recall of serial order information was more accurate when, during the encoding phase, the WM items progressed from left to right compared to situations where they progressed in a right to left fashion.

More broadly, it may be interesting to note that our account corresponds with specific aspects of memory research in general. Most notably, the mnemonic tool referred to as the method of loci [35, 36] builds on the idea that memory performance can be facilitated by visualizing to-be-remembered items in a familiar scene which you mentally walk through during item retrieval. Hence, like in our account, (working) memory performance is modulated by the use of spatial organization. Future work should explore the possibility of spatial coding of serial order WM as a determinant underlying the success of the method of loci, or whether this link merely involves an illustrative analogy.

In the current study we tested and confirmed a clear prediction from the mental whiteboard hypothesis. Yet, it may be argued that the observed link between serial order WM and spatial processing might be the *indirect* result of the well-established link between number and space processing. Specifically, serially presented items may be tagged to fixed number codes to maintain serial order—for example, a first item is tagged to the representation of “1” or “first’ in, a second item to “2” or “second”, etc [2, 6]. These order tags, then, may subsequently drive spatial processing in line with the Spatial Numerical Association of Response Codes or SNARC effect [37]. While we cannot entirely reject this alternative mechanism, the findings of van Dijck et al. ([15, 17], see also [18]) show that the spatial coding of serial order in WM occurs in the absence of any magnitude-based spatial priming (despite indications that the numerical magnitude was processed). These (and other) observations led us to claim in previous work that spatial effects of number magnitude may be driven by serial order WM [17, 38]—and not the other way around. Future research will need to further establish this primacy of serial order WM over number magnitude effects.

Finally, a closer look at the data of Experiment 2 learns that the spatial cueing effect for the fourth WM position is numerically smaller than would have been expected on the basis of the regression line. While we have no immediate and conclusive explanation for this observation, it should be noted that it is not the first time this observation is made (e.g., [17]). Further research is needed to understand the exact reason for the attenuated effect at position four, and why this is only observed in some studies.

Overall, the current findings indicate that verbal serial order WM and space are intrinsically related to each other by pointing to the existence of a bidirectional link. This observation is strong empirical evidence for the idea that serial order coding occurs within a spatially defined coordinate system, as proposed in our mental whiteboard hypothesis [9]. Future studies will be needed to further explore exact underlying cognitive and brain processes and to further challenge verbal serial order coding theories to consider spatial processes as a core ingredient.

## Supporting Information

S1 Dataset Data of Experiment 1. (XLSX)

S2 Dataset Data of Experiment 2. (XLSX)
