# Correction: Serial Position Markers in Space: Visuospatial Priming of Serial Order Working Memory Retrieval

**DOI:** 10.1371/journal.pone.0214762

**Published:** 2019-03-28

**Authors:** Maya De Belder, Elger Abrahamse, Mauro Kerckhof, Wim Fias, Jean-Philippe van Dijck

## Notice of Republication

This article was republished on March 20, 2019, to correct the author list. Please download this article again to view the correct version.
